# Tumor Initiating Cells and Chemoresistance: Which Is the Best Strategy to Target Colon Cancer Stem Cells?

**DOI:** 10.1155/2014/859871

**Published:** 2014-01-15

**Authors:** Emanuela Paldino, Valentina Tesori, Patrizia Casalbore, Antonio Gasbarrini, Maria Ausiliatrice Puglisi

**Affiliations:** ^1^Institute of Cellular Biology and Neurobiology, National Research Council, Via del Fosso di Fiorano 64, 00143 Rome, Italy; ^2^Department of Internal Medicine and Gastroenterology, Gemelli Hospital, Largo Agostino Gemelli 8, 00168 Rome, Italy

## Abstract

There is an emerging body of evidence that chemoresistance and minimal residual disease result from selective resistance of a cell subpopulation from the original tumor that is molecularly and phenotypically distinct. These cells are called “cancer stem cells” (CSCs). In this review, we analyze the potential targeting strategies for eradicating CSCs specifically in order to develop more effective therapeutic strategies for metastatic colon cancer. These include induction of terminal epithelial differentiation of CSCs or targeting some genes expressed only in CSCs and involved in self-renewal and chemoresistance. Ideal targets could be cell regulators that simultaneously control the stemness and the resistance of CSCs. Another important aspect of cancer biology, which can also be harnessed to create novel broad-spectrum anticancer agents, is the Warburg effect, also known as aerobic glycolysis. Actually, little is yet known with regard to the metabolism of CSCs population, leaving an exciting unstudied avenue in the dawn of the emerging field of metabolomics.

## 1. Introduction

Colorectal cancer (CRC) is the third most common type of cancer and the second leading cause of tumor related death in the western world [1]. Despite the well-known genetic mutations that drive the transition from healthy colonic epithelia to dysplastic adenoma and finally to colon adenocarcinoma, current anticancer treatments are often able to eradicate the disease. Indeed, the response rate to current systemic therapies is of *∼*50%, but resistance develops in nearly all patients [2].

Conventional first-line treatments for patients with metastatic CRC to the liver involve a combination of 5-fluorouracil, leucovorin, and oxaliplatin (FOLFOX) and a combination of 5-fluorouracil, leucovorin, and irinotecan (FOLFIRI). FOLFOX and FOLFIRI have demonstrated good efficacy in phase III trials and are actually employed in younger patients with metastatic CRC [3]. Moreover, neoadjuvant chemotherapy has been combined with antiangiogenic drugs, particularly with bevacizumab (Avastin), which target vascular endothelial growth factor (VEGF), and cetuximab (Erbitux) that inhibits the epidermal growth factor receptor (EGFR) [4, 5]. Although these types of combination therapies have increased disease-free survival and improved overall survival in patients with CRC, most patients with metastatic disease are not cured.

Therefore, a better understanding of the resistance mechanisms is essential as a first step in developing approaches to prevent or reverse chemoresistance in patients who receive systemic therapy for metastatic CRC. There is an emerging body of evidence that tumor cells that are resistant to chemotherapy represent a subpopulation of cells from the original tumor that are molecularly and phenotypically distinct. These cells are called tumor initiating stem cells or cancer stem cells (CSCs). Currently, it is believed that only this small fraction of cancer cells is able to drive tumor initiation, proliferation, and spreading [6]. Biologically distinct populations of CSCs have been identified in most solid tumors, including colon cancer [7, 8].

CSCs are characterized by three unique properties: the capacity of self-renewal, the ability to recreate the full repertoire of cancer cells of the parental tumor, and the expression of a distinctive set of surface biomarkers [9].

CSCs can divide to yield a more differentiated cell and a daughter cell that maintains the same properties as the parental cell. This ability of self-renewal enables CSCs to perpetuate the tumor [6]. Actually, despite their capacity for self-renewal, CSCs are relatively quiescent; that is, they have low proliferative rate and are often not cycling. Indeed, they have been shown to have significantly longer cell cycle times than proliferating nonstem cells (SCs). This is presumably due to the arrest of CSCs at a G0-like cell cycle phase or checkpoint [10].

CSCs are also identified from the expression of one or multiple cell surface markers associated with cancer stemness, such as CD133 [7, 8, 11], CD44 [12–14], CD24 [15], CD166 [14], and Lgr5 [16]. More functional markers such as Wnt activity [14] and ALDH1 activity [17] have been exploited for identification of colon CSCs. However, none of the markers used to isolate stem cells in various cancerous tissues are expressed exclusively by the stem cell fraction. Indeed most of the markers used for colon CSCs isolation are chosen either because they are expressed in normal stem cells or as they were found to identify CSCs in other malignancies.

Cancer stem cells theory has profound translational implications. Antitumor therapies that do not target CSCs may lead to a reduction of the tumor mass but not the regression of the tumor. In fact, therapies are generally based on drugs that affect rapidly dividing cells, while CSCs display low proliferative potential [18]. Furthermore, CSCs are relatively resistant to cytotoxic systemic therapies [2]. Indeed, CSCs display alterations of DNA repair, due to the presence of cytoprotective properties (including telomerase activation and high expression of antiapoptotic factors) and express high levels of proteins belonging to the ABC membrane transporters family, involved in chemotherapeutic resistance [19]. Thus, many tumors may progress because CSCs are not sensitive to the treatment.

Novel therapies should be developed to target CSCs [19]. These include induction of CSCs differentiation or targeting genes that control self-renewal and chemoresistance in CSCs.

## 2. Differentiation Therapy to Target the CSCs

The differentiation induction is one of the therapeutic options proposed to eliminate or functionally antagonize CSCs. This therapeutic strategy consists in forcing CSCs to shift into a terminal epithelial phenotype, losing their self-renewal abilities, and therefore becoming vulnerable to conventional therapies. Even if this approach does not directly kill CSCs, it could increase the efficiency of conventional therapies in eradicating the tumor. A well-established example of differentiation therapy is the use of all-trans retinoic acid in combination with chemotherapy in the treatment of acute promyelocytic leukemia [20].

Recently, the use of bone morphogenetic protein 4 (BMP4) has been proposed to induce differentiation in colon CSCs [21]. Indeed, BMP4 is able to activate a differentiation program and stimulate apoptosis in colon CSCs, reducing *β*-catenin activation through inhibition of PI3K/AKT pathway and upmodulation of Wnt-negative regulators [21].

Bone morphogenetic proteins are important players in the differentiation program of the normal gut. These cytokines act by defining a decreasing gradient from the intestine lumen toward the crypt, counteracting stem cell expansion outside the crypt, and promoting intestinal epithelial cell differentiation [22, 23]. Loss of these homeostatic mechanisms may contribute to tumor initiation [23].

In recent studies Lombardo and colleagues [24] have demonstrated that BMP4 expression is limited to the differentiated progeny of CRC epithelial cells, which constitute the major population of the tumor mass. Instead, BMP4 is undetectable in the colon CSCs fraction.

Authors have showed that the exposition to BMP4 could activate BMP canonical signaling pathway promoting a rapid and massive terminal differentiation, apoptosis, and chemosensitization of colon CSCs. Indeed, exogenous administration of BMP4 to immunocompromised mice with tumors, which arose from colon CSCs, increased the antitumor effects of 5-fluorouracil and oxaliplatin, confirming that BMP4 might be developed as a therapeutic agent against cancer stem cells in advanced colorectal tumors [24]. Although the clinical use of such a combination would require the improvement of BMP4 delivery and optimization of the treatment schedule, the considerable enhancement of the therapeutic response obtained in mouse xenografts, even in long-term experiments, supports further investigations toward the use of differentiation therapy in CRC.

More recently, it was reported that BMP signaling can inhibit Wnt activity, which functionally designates the colon CSCs, in mouse models resulting in suppressed crypt formation and reduced polyp growth [25]. Furthermore, an ever-growing number of studies have recently suggested that microRNAs are functionality implicated in chemoresistance processes [26, 27].

MicroRNAs are endogenous posttranscriptional modulators, whose dysregulation plays an important role in the development and progression of many malignancies, including colorectal carcinoma. In particular, Yu and colleagues [28] have demonstrated that the expression of microRNA-21 (miR-21), whose levels are significantly increased in chemotherapy-resistant colon tumors, is also highly elevated in colon CSCs. Authors hypothesized that miR-21 could play a critical role in regulating differentiation of colon CSCs. Indeed, they reported that the downregulation of miR-21 in colon cancer cell lines (HCT-116 or HT-29), through transfection of antisense miR-21, induced differentiation, as evidenced by increased expression of gastrointestinal differentiation marker cytokeratin-20 (CK-20) [29] and an increase in alkaline phosphatase activity [30]. These changes were also accompanied by significantly decreased expression levels of colon CSCs marker CD44 [31] and epidermal growth factor receptor (EGFR), reduction of colon-sphere formation, and increased expression of proapoptotic genes [28]. Moreover, recent study showed that downregulation of miR-21 made colon CSCs greatly susceptible to conventional or nonconventional chemotherapeutics, such as 5-fluorouracil + oxaliplatin (FUOX), difluorinated curcumin (CDF), and the combination of CDF and FUOX [28].

Overall these results illustrate the possibility to establish a roadmap of the signaling pathways that control CSCs tumorigenicity, allowing the identification of specific molecular targets for future colon cancer therapy.

## 3. Blockade of Epithelial to Mesenchymal Transition

Epithelial to mesenchymal transition (EMT) is a transdifferentiation process by which epithelial cells shed their epithelial characteristics and acquire migratory mesenchymal cell-like properties. Several key signaling pathways contribute to this process, such as transforming growth factor-*β* (TGF-*β*) and Wnt, whose activities are dysregulated during malignant tumor progression. Thus, EMT induction in cancer cells results in the acquisition of invasive and metastatic properties [32, 33]. Moreover, EMT complicates therapeutic approaches because it participates in the acquisition of both de novo and acquired drug resistance [34]. Indeed, EMT can trigger reversion to a CSC-like phenotype, providing an association between EMT, CSCs, and drug resistance [35]. Targeting EMT pathways and CSCs maintenance might be a promising therapeutic strategy.

Qi and colleagues have demonstrated the ability of Dickkopf-1 (Dkk1), a potent Wnt signaling inhibitor, to reverse EMT in colon cancer [36]. Authors, analyzing 217 patients affected by colon cancer, found that Dkk1 expression was inversely correlated with tumor stage, the presence of metastasis, and recurrence. In particular, they observed that Dkk1-positive samples displayed higher expression level of epithelial marker E-cadherin and decreased expression of mesenchymal marker vimentin and cytoplasmic distribution of b-catenin than in Dkk1-negative samples, suggesting that the loss of Dkk1 in colon cancer could contribute to its progression and that Dkk1 was possibly associated with a reversal of the EMT process [36]. Furthermore, Dkk1 overexpression in HCT-116 colon cancer cell line resulted in restoration of the epithelial phenotype, decreased expression of Snail and Twist, two transcription factors considered to be key regulators of EMT [37], and decreased expression of colon CSCs markers (such as CD133 and leucine-rich-repeat-containing G-protein-coupled receptor 5 (Lgr5)). Functional analysis showed that upregulation of Dkk1 led to decreased tumor initiating ability and suppressed colon tumor growth in nude mice, suggesting that Dkk1 could suppress the progression of colon cancer, possibly through EMT inhibition, and could therefore serve as a target for tumor therapy [36].

These findings may be important for future studies on the mechanism of colon cancer progression and for effective targeting of EMT via Dkk1, which may offer new hope for anticancer therapy.

Several studies have also suggested that small molecule inhibitors of TGF-*β* may be a useful therapeutic tool for the treatment of metastatic colon cancer [38].

Indeed, TGF-*β* is a potent inducer of EMT, directly activating the expression of transcription factors such as SNAI1/2, Twist, and ZEB1/2, which are the key regulators of the EMT program [39]. Thus, endogenous TGF*β* signaling is associated with poor metastatic outcome in colon cancer, and deregulated TGF*β* signaling correlates with tumor development and metastasis.

Recent studies have demonstrated that synthetic peptides, called P17 and P144, previously characterized as inhibitors of TGF-*β*1 [40], could be a new possible therapy to impair colon cancer-derived liver metastasis [41, 42]. In particular, the combination of immunotherapeutic strategies with peptide inhibitors of TGF-*β* was able to enhance the efficacy of immunotherapy, suggesting that these compounds may be useful for future clinical application in cancer immunotherapy [41].

More recently, Zubeldia and colleagues have demonstrated that the injection of colon adenocarcinoma cells expressing luciferase, pretreated with TGF-*β* (Mc38-luc TGF-*β*1), into the spleen of mice, increased primary tumor growth and liver metastasis. On the other hand, systemic treatment of mice with either P17 or P144 significantly reduced tumor burden (*P* < 0.01) [43]. Authors observed that in metastatic nodules, mitotic/apoptotic ratio, mesenchymal traits, and angiogenesis induced by TGF-*β* were consistently reduced following injection of peptides. In vitro experiments revealed a direct effect of TGF-*β* in Mc38 cells, which resulted in activation of Smad2, Smad3, and Smad1/5/8, and increased invasion and transendothelial migration, whereas blockade of TGF*β*-signaling reverted these features. Finally, authors demonstrated that TGF-*β* treated cells displayed a greater capacity of tumor-sphere formation, which were also enriched in CD44 and SOX2. This ability was significantly diminished in the presence of P17, providing a preclinical rationale to evaluate P17 and P144 as potential therapeutic options for the treatment of metastatic CRC [43].

## 4. Target Specific CSCs Pathways

Advances in high-throughput technologies and bioinformatics will allow developing additional compounds specifically targeting CSCs signaling pathways. Currently there are two established targets for such therapies: EGFR, which belongs to the ErbB family of tyrosine kinase receptors and is abnormally activated in many tumors [44], and VEGF, which is known to promote formation of new vessels by inducing growth and differentiation of endothelial cells [45, 46]. Several clinical trials have demonstrated that introduction of targeted therapies with monoclonal antibodies against EGFR (cetuximab) and VEGF (bevacizumab) in addition to 5-FU resulted in a significant survival increase in patients with advanced disease [47].

Another CSCs target includes blockage of various self-renewal pathways, including Wnt, Notch, PTEN, and Hedgehog [48]. Small molecules that inhibit the Wnt pathway and *γ*-secretases that inhibit the Notch pathway have been recently identified as novel approaches to CRCs [49]. The Wnt/*β*-catenin pathway has been implicated in the maintenance of the intestinal crypt stem cell phenotype, and Wnt signaling dysregulation through either loss of APC function or oncogenic *β*-catenin mutations has been shown to cause the majority of sporadic cancer cases [50]. Disruption of Tcf/*β*-catenin complexes by selected small molecule antagonists has been shown to antagonize cellular effects of *β*-catenin and to result in inhibition of cellular proliferation in colon cancer cells [51]. Similarly, the Notch signaling pathway has been reported to be overexpressed in colon CSCs, where it was found to play a role in colon CSCs viability, tumorigenicity, and self-renewal [52, 53]. van Es and colleagues have demonstrated that blocking the Notch cascade with a gamma-secretase inhibitor induced goblet cell differentiation in adenomas, in mice carrying a mutation of the Apc tumor suppressor gene, and subsequent tumor growth arrest [54]. Moreover, Hoey et al. have demonstrated that by inhibiting delta-like 4 ligand (DLL4), an important component of the Notch pathway, with human monoclonal antibody 21M18 in colon carcinoma xenografts, the tumor growth as well as the CSCs frequency was decreased compared to control. Interestingly, even though treatment of the xenografts with irinotecan, a chemotherapeutic often used in colon cancer, slowed down tumor growth, the clonogenicity was increased. Combination treatment of irinotecan with anti-hDLL4 reduced again the tumor growth and stem cell frequency, at even higher levels than the anti-DLL4 treatment alone [55]. This indicates that inhibiting Notch signaling reduces CSCs frequencies and sensitizes tumor cells for irinotecan treatment.

It has recently been observed that the inhibition of the IL-4 pathway with an anti-IL-4 antibody or an IL-4 receptor antagonist in CD133+ colorectal CSCs augmented the antitumor effects of conventional chemotherapeutics [56, 57]. Indeed, colon carcinomas produce IL-4 that acts in an autocrine manner, promoting antiapoptotic pathways in these tumors. The IL-4 inhibition by blocking antibodies sensitizes the cells for killing by 5-FU and oxaliplatin IL-4 [56, 57].

## 5. Metabolic Target Strategy

The recent cluster analyses of driver mutations have showed that several of these mutations converge on metabolic pathways [58–62], revealing that cancer hallmarks can indeed impinge on cancer metabolism. Actually, little is yet known with regard to the metabolism of CSCs population, leaving an unstudied exciting avenue in the dawn of the emerging field of metabolomics.

A recent study by Akao and colleagues provided initial evidence of metabolic changes in therapy-resistant cell populations, by demonstrating significant overexpression of a metabolic “master-regulator,” Sirt1 (Silent mating type information regulation 1), in human colorectal cancer DLD-1 5-FU-resistant cells [63]. Sirt1 plays a crucial role in various cellular processes including senescence and cell survival under genotoxic and oxidative stresses [64, 65]. Moreover, Sirt1 has been implicated in the promotion of tumorigenesis and development of drug resistance. Authors have demonstrated that silencing of Sirt1 significantly abrogated the resistance to 5-FU in the 5-FU-resistant cells, suggesting that targeting the Sirt1 gene could negatively regulate, at least in part, the resistance to 5-FU in human colorectal cancer [63].

The Warburg effect, also known as aerobic glycolysis, is another important aspect of cancer biology that can be harnessed to create novel broad-spectrum anticancer agents [66–68]. The Warburg effect is defined as the propensity of cancer cells to take up high levels of glucose and to secrete lactate in the presence of oxygen and is linked to oncogenic transformation in a manner that frequently implies the inactivation of metabolic checkpoints, such as the energy rheostat AMP-activated protein kinase (AMPK) [69].

Recently, metformin has emerged as anticancer agent via AMPK inhibition [70]. Epidemiological, preclinical, and clinical evidence supports the use of metformin as a cancer therapeutic. In particular, several studies have demonstrated the ability of metformin for the treatment of cancers known to be associated with hyperinsulinemia, such as breast and colon cancers [71–73].

Interestingly, metformin may selectively kill cancer stem cells, since increased dependency on Warburg-like aerobic glycolysis (hyperglycolytic phenotype) is critical to sustain CSCs stemness and immortality. An interesting study conducted by Hirsch et al. has demonstrated the effect of metformin on breast CSCs [74]. Metformin treatment was found to specifically eliminate CD44^+^/CD24^−/low^ CSCs and had a synergistic effect with doxorubicin, which resulted in reduced tumor burden and delayed tumor recurrence [74]. Furthermore, metformin combined with doxorubicin/cisplatin or paclitaxel delayed tumor relapse better than either agent alone. Surprisingly, the combination was effective with a fourfold lower dose of doxorubicin, thus decreasing the toxicity of chemotherapy and improving its efficiency. Moreover, in highly metastatic basal-like MDA-MB-231 breast cancer cells, has been showed that metformin treatment dynamically suppresses the CD44^+^/CD24^−/low^ CSCs phenotype via transcriptional repression of the EMT machinery including transforming growth factor-*β*1, ZEB, Twist, and SLUG (Snail2) [75].

In a recent study Menendez and colleagues hypothesize that exogenous microenvironmental factors (e.g., hypoxia, oxidative stress, extracellular matrix detachment, and nutrient starvation) and intrinsic genetic determinants (e.g., oncogenes, tumor suppressor genes, and epigenetics) could converge to coordinately determine the activation of the Warburg phenotype in tumor tissues. In this state, tumor cells may adapt a Warburg phenotype and CSCs cellular states should have a powerful growth and survival advantage. Authors proposed that the Warburg effect could permit reprograming the tumor cells of origin, so that they can acquire the cellular state of a CSC, allowing the conversion of non-CSCs into CSCs [76].

This inherent plasticity of the CSCs phenotype implies that eliminating CSCs alone may not effectively cure tumors, as they can be regenerated from non-CSCs, calling for dual targeting therapeutic regiments. The identification of the metabolic infrastructure and the metabolic functioning of CSCs could be the basis to novel therapeutic approaches.

## 6. Conclusion

Altogether, these data illustrate the therapeutic utility of the cancer stem cell concept, which provides the tools for discovery of novel mechanisms of cancer therapeutic resistance. Inhibitors of survival pathways, along with differentiation agents and cytotoxic drugs, might be used in combination to treat patients with metastatic colon cancer. Interestingly, the opportunities and challenges for targeting the metabolic infrastructure of CSCs might be rapidly achieved, because existing metabolic drugs may be easily repositioned from preclinical stages to clinical approaches.
